# 

**Published:** 1851-03

**Authors:** 


### CONTENTS:

### PART I.—ORIGINAL COMMUNICATIONS.

ART. 1.—On the use of Chloroform as a Therapeutical Agent, by Dr. James Smick. - - - - - - - 409
" 2.—Medical Topography of York Township, Michigan, by Dr. W. W. Goff. - - - - - - - 415
" 3.—Abnormal Developement of the Liver and Spleen at birth, by D. Hutchinson, M. D. - - - - - - 419
" 4.—Hæmatocele, by Dr. R. W. Hall. - - - - - 420
" 5.—Collodion in Mammary Inflammation and Small Pox, by Dr. J. H. Murphy. - - - - - - - 423
" 6.—Clinical Lectures and cases in the Wards of Illinois General Hospital, by Prof. N. S, Davis. - - - 425
" 7.—Collodion in Chilblains, by Prof. John Evans. - - - 433
" 8.—Inoculation in Rubeola, by John E. McGirr, A. M., M. D., L. L. D. - - - - - - 434
" 9.—A Family Poisoned by eating a Garr, by Dr. W. Brooks, 437
" 10.—History of Cholera in the vicinity of Victoria, Knox Co. Ill. by J. W. Spalding. - - - - - 429

### PART II.—REVIEWS AND NOTICES OF NEW WORKS.

ART. 1.—Transactions of the Amer. Med. Ass., for 1850. - - 441
" 2.—Southern Medical Reports (concluded.) - - - 451
" 3.—Anniqersary Discourse before the N. Y. Academy of Mediicine, by J. M. Smith, M. D.,—Address delivered before the Students and Trustees of N. Y. Medical College, by Horace Green, A. M., M. D.,—Introductory Address to class of Penn. College, by Wash. L. Atlee, M. D. - - - - 461
" 4.—Curability of Consumption considered, by M. Mattson, M. D. 462
" 5.—Ranking's Half-Yearly Abstract, for December. - - 462

### PART III.—SELECTIONS.

ART. 1.—The Spirometer. - - - - - - - 463
" 2.—Dr. Davis' "History of Medical Education and Institutions," and the Reviewer in the N. Y. Journal of Medicine. - - 469
" 3.—California Quicksilver. - - - - - 471
" 4.—Valedictory of St. Louis Probe. - - - - 472
" 5.—On treatment of Scarlatina by Inunction, by Dr. Sehneeman. 473
" 6.—Midwifery and Diseases of Women. - - - - 478
" 7.—Venereal Affections. - - - - - 480
" 8.—The Salts of Morphia. - - - - - - 482
" 9.—Analysis of Prairie Soil. - - - - - - 482
" 10.—New Method of operating for Radical Cure of Inguinal Hernia, by M. Varrett. - - - - - - - 485
" 11.—On treatment of Bronchocele, by Compression, by Wm. C. Dwight, M. D. - - - - - - - 487
" 12.—Cholera at Jamaica. - - - - - - 488
" 13.—American Medical Association. - - - - 489
" 14.—Collodion in Erysipelas. - - - - - 489

### PART IV.—EDITORIAL.

ART. 1.—Valedictory. - - - - - - - 491
" 2.—Illinois General Hospital. - - - - - 492
" 3.—Medical Societies. - - - - - - 494
" 4.—Prof. Herrick's Valedictory Address to Graduating Class in Rush Medical College. - - - - - 496
" 5.—American Medical Association. - - - 496
" 6.—New Medical Journals. - - - - 497
" 7.—Miscellaneous Medical Intelligence. - - 497
" 8.—Obituary. - - - - - - - - 500
" 9.—Important Erratum. - - - - - 500

